# Automated pose estimation reveals walking characteristics associated with lameness in broilers

**DOI:** 10.1016/j.psj.2023.102787

**Published:** 2023-05-19

**Authors:** István Fodor, Malou van der Sluis, Marc Jacobs, Britt de Klerk, Aniek C. Bouwman, Esther D. Ellen

**Affiliations:** ⁎ Animal Breeding and Genomics, Wageningen University & Research, 6700 AH Wageningen, the Netherlands; † FR Analytics B.V., Wierden, the Netherlands; ‡ Cobb Europe, Boxmeer, the Netherlands

**Keywords:** computer vision, gait score, broiler, DeepLabCut, precision phenotyping

## Abstract

Walking ability of broilers can be improved by selective breeding, but large-scale phenotypic records are required. Currently, gait of individual broilers is scored by trained experts, however, precision phenotyping tools could offer a more objective and high-throughput alternative. We studied whether specific walking characteristics determined through pose estimation are linked to gait in broilers. We filmed male broilers from behind, walking through a 3 m × 0.4 m (length × width) corridor one by one, at 3 time points during their lifetime (at 14, 21, and 33 d of age). We used a deep learning model, developed in DeepLabCut, to detect and track 8 keypoints (head, neck, left and right knees, hocks, and feet) of broilers in the recorded videos. Using the keypoints of the legs, 6 pose features were quantified during the double support phase of walking, and 1 pose feature was quantified during steps, at maximum leg lift. Gait was scored on a scale from 0 to 5 by 4 experts, using the videos recorded on d 33, and the broilers were further classified as having either good gait (mean gait score ≤2) or suboptimal gait (mean gait score >2). The relationship of pose features on d 33 with gait was analyzed using the data of 84 broilers (good gait: 57.1%, suboptimal gait: 42.9%). Birds with suboptimal gait had sharper hock joint lateral angles and lower hock-feet distance ratios during double support on d 33, on average. During steps, relative step height was lower in birds with suboptimal gait. Step height and hock-feet distance ratio showed the largest mean deviations in broilers with suboptimal gait compared to those with good gait. We demonstrate that pose estimation can be used to assess walking characteristics during a large part of the productive life of broilers, and to phenotype and monitor broiler gait. These insights can be used to understand differences in the walking patterns of lame broilers, and to build more sophisticated gait prediction models.

## INTRODUCTION

Impaired walking ability is a welfare concern in broiler production (Knowles et al., 2008). Broiler walking ability is commonly assessed by a manual gait scoring system, for example, that of Kestin et al. (1992). With this system, experienced scorers observe individual birds while walking and grade their walking ability along a 6-point scale, with a gait score (**GS**) of zero representing birds that walk normally and a GS of 5 representing birds that are incapable of sustained walking on their feet. Impaired walking ability has a high prevalence in broiler production. For example, a UK survey indicated that 27.6% of birds show poor locomotion (GS3 or higher), with 3.3% being almost unable to walk (Knowles et al., 2008).

Impaired walking ability may result in discomfort and behavioral changes. For example, Riber et al. (2021) showed that broilers with impaired walking ability (GS2) were less likely to perch, spent more time sitting while feeding, and spent less time performing comfort behavior (i.e., preening, wing flapping, stretching legs or wings, feather ruffling/shaking) than birds walking normally (GS0). Moreover, a tendency was observed for broilers with impaired walking ability to spend more time inactive (Riber et al., 2021). Together this highlights that impaired walking ability is a welfare concern. Lameness also leads to substantial economic losses on broiler farms, negatively affecting daily weight gain, feed conversion ratio, mortality rate, and condemnation rate (Gocsik et al., 2017).

It appears that there is potential for selection against (different components of) impaired walking ability. For example, it has been shown that tibial dyschondroplasia and hock burn are heritable in broilers, with heritability estimates of 0.10 to 0.27 and 0.06 to 0.09, respectively (Kapell et al., 2012). However, to breed for improved walking ability or leg health, individual-level phenotypic records are required for many birds. As mentioned earlier, walking ability is often scored manually, mainly through visual gait scoring approaches (e.g., Kestin et al., 1992; Garner et al., 2002; Dawkins et al., 2004; Webster et al., 2008), but also a shallow water or latency to lie test can be used (Weeks et al., 2002). However, these manual approaches are time-consuming and laborious.

Automated approaches for scoring gait could be of great added value. Several automated approaches have already been developed, some of which assess proxies for gait, while others measure walking characteristics. Cameras have been used to record proxies for gait, such as activity (Dawkins et al., 2009; Aydin et al., 2013; Silvera et al., 2017) or lying behavior (Aydin, 2017b). Another option is to use body-worn sensors, for example, to record activity using ultra-wideband technology (van der Sluis et al., 2021), or to follow the broilers’ behavior using accelerometers (Abdoli et al., 2018). Walking characteristics in poultry have been analyzed using kinetic sensors, for example, a pedobarograph (Corr et al., 1998) or a force plate (Corr et al., 2007), however, computer vision-based solutions have been gaining popularity. Video recordings have been made of birds walking along a walkway or corridor, which were subsequently analyzed using image analysis to extract features such as speed, step frequency, step length, and lateral body oscillation (Aydin, 2017a). Features describing walking were used in a decision tree classification approach (de Alencar Nääs et al., 2021), whereas others detected and tracked keypoints on the bodies of walking broilers as input for pose estimation-based models (Nasiri et al., 2022), to predict lameness.

There are several challenges when implementing these sensor-based approaches for larger-scale automated gait scoring. For example, birds need to be individually handled to record the data (e.g., Aydin, 2017a), which can be time-consuming and provides only a single time point record. Other methods can record data at the individual level automatically over time, but might require a sensor per animal (e.g., van der Sluis et al., 2021). There appears to be potential to implement pose estimation-based methods for automated gait scoring without handling the birds or attaching sensors, with good results (Nasiri et al., 2022), but using a black box approach in which it remains unknown which exact pose features correlate with broiler gait scores. There would be added value in gaining insight in the walking characteristics of broilers that underlie their gait scores.

In the current study, it was investigated whether specific walking characteristics of broilers could be determined through automated pose estimation and could be linked to broiler gait scores. Using back-view video recordings of broilers walking, keypoints on the broilers’ bodies were extracted for automated pose estimation of 7 features of broilers with either a good or a suboptimal gait. The results of this study provide valuable insights into the walking characteristics of broilers with a good or suboptimal gait and can, although currently tested in a small-scale setting using a walkway, contribute to future automation of gait scoring in broilers.

## MATERIALS AND METHODS

### Ethical Statement

Data were collected on a broiler farm in the Netherlands, under control of Cobb Europe (Boxmeer, the Netherlands). Cobb Europe complies with the Dutch legislation on animal welfare.

### Data Acquisition

**Figure 1:**
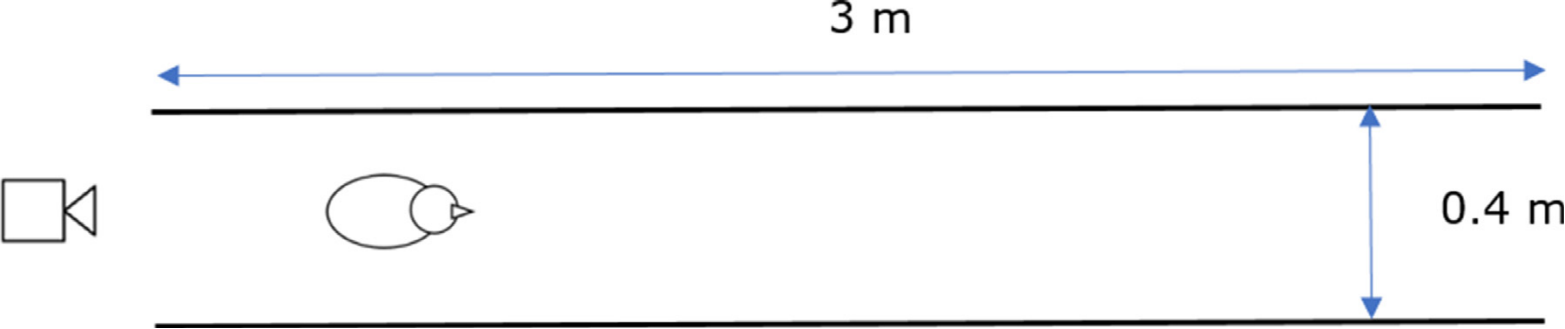
Setup of the walking trial.

Individually tagged male broilers from the same cross were filmed 3 times, at 14, 21, and 33 d of age, at a Cobb test facility in the Netherlands. The broilers were group-housed, with ad libitum access to feed and water, and wood shavings as bedding. Commercial lighting and temperature schedules were used, and vaccination was performed according to common practice (Cobb, 2018). A total of 109 birds, of 14-days old, were housed in 2 pens at the start of the trial. By 21 and 33 d of age, 108 and 87 birds remained for video recording, respectively. The major reason for removing birds from the study was routine activities of the company for the breeding program. For this purpose, all birds within a pen were weighed individually, to calculate the descriptive statistics of body weight per pen. Thereafter, individual birds (*n* = 6 per pen, 12 birds in total) representing the mean body weight, mean ± 1*SD, and mean ± 2*SD within the pen were identified based on a list, and subsequently removed from the pen. A few birds (*n*_D14–D21_ = 1, *n*_D21–D33_ = 9) were removed due to mortality. On each day of recording, individual body weight of the birds was measured before the walking trial. During the walking trial, birds had to walk through a corridor created within their pen one by one, with dimensions 3 m × 0.4 m (length × width, Figure 1). An Intel RealSense D415 (Intel Corp., Santa Clara, CA) camera was placed at bird level in the midline of the start of the corridor, which recorded the birds from behind during walking. The RGB video recordings of the camera were converted to .mp4 format with 12 frames per second and 1,280 × 720 pixels resolution for further analysis using a custom Python code. Start and end times were manually recorded for each individual, and only the video frames in this period were used for further analyses.

### Keypoint Detection Using Convolutional Neural Network

Eight keypoints (head, neck, left and right knees, hocks, and feet) were detected using a pretrained broiler pose estimation deep learning model, developed in DeepLabCut (Mathis et al., 2018). Details of the pretrained broiler pose estimation model are described in Doornweerd et al. (2021). Briefly, the convolutional neural network (**CNN**) was trained on 1,224 frames of 37 broilers, recorded from behind at 37 d of age, while completing an individual walking test in a 3 m × 0.4 m corridor. Their setup was very similar to the one used in the current study, with the same camera and viewpoint, and the same nontransparent side panels to create the corridor, although, the recording took place on another farm. Keypoints were annotated at the following locations: the head at the top, the neck at the base, the knees at the estimated location of the knee, the hocks at the transition of the feathers into scales, and the feet in the middle at the approximate height of the first toe (Doornweerd et al., 2021). Model performance was tested on 306 frames of 10 birds. The train and test errors of this broiler model were 2.26 and 7.56 px, respectively, on the keypoints that surpassed the likelihood threshold of 0.6 (the default in DeepLabCut).

However, this pretrained model struggled with accurately detecting the keypoints of the broilers recorded in the new environment. The major differences in our setup were the following: 1) illumination was different on this farm, 2) red drinkers were present at the drinking line, above the head of the broilers, 3) after completion of the walking trial, some broilers stayed at the end of the corridor standing or resting, and 4) birds were recorded already from 14 d onward, having a considerably smaller size than 37-day-old broilers. The main issues observed were jumping keypoint labels between the bird of interest and birds staying at the end of the corridor at 14 d of age, keypoint detections jumping between the head of the bird and the red drinker above their head, and missing keypoint detections on the legs at later ages due to the relatively darker circumstances. To overcome these issues, we first adjusted the brightness of 40% of the training frames randomly within a range of ±30%, augmented the training data with these frames, and applied dynamic cropping. Dynamic cropping computes object boundaries according to the smallest and largest *x* and *y* coordinates of all detected keypoints in a frame, which is further expanded by a margin, and only the posture within this crop is further analyzed. The current position of the object is used to update the crop window in the next frame. These steps did not substantially improve model performance, so we decided to further train the model of Doornweerd et al. (2021). Therefore, video frames of 17 birds that had been removed during our study between d 14 and 33 were used for retraining of this model, hereby avoiding further decrease of our sample size. Altogether, 181 frames were manually selected from videos of 5 broilers from d 14 and 8 broilers from d 21 for further training of the model, whereas 40 frames (2 broilers from d 14 and d 21, respectively) were used for testing the retrained model, resulting in a 82/18 train-test split. Annotations were performed as described in Doornweerd et al. (2021). The aim of the manual frame selection was to further train the model specifically on those situations that were challenging for the pretrained model in the new environment. This model was then used for the analysis of the newly acquired videos, along with a spline filter and dynamic cropping (the latter in d 14 videos only, as label jumping between birds was mostly observed at this age) to reduce noise in the keypoint estimates. The performance of the retrained broiler pose estimation deep learning model was assessed using the pixel error, which is the Euclidean distance between the *x* and *y* coordinates of the model predictions and the human annotator (Doornweerd et al., 2021). The percentage of keypoints exceeding a likelihood threshold of 0.6 by age and body part was also assessed for the analyzed videos. These percentages reflect the certainty of all keypoint detections in the full length of the analyzed videos, and include, for example, the seconds between the start of recording and placing the bird in the corridor. Coordinates (*x, y*) and likelihoods of the estimated keypoints from the newly acquired videos were downloaded in .csv format and used for pose extraction and calculating the features.

### Automated Pose Extraction

Birds that did not walk during the test (*n*_D21_ = 1, *n*_D33_ = 3) were excluded from further analyses, because it was not possible to obtain reliable keypoints and poses for them. We decided not to base the exclusions on the gait scores, to be able to characterize birds with very poor gait if the model could capture their keypoints and poses.

We had 2 poses of interest: double support, when broilers are standing with both feet on the ground, and steps at maximum leg lift (left and right steps). The first and last 10% of frames of each walking trial were discarded. The first 10% mostly did not contain the bird of interest or showed the moment when the bird was placed in the corridor. Based on visual assessment, during the last 10% the bird of interest was more difficult for the model to distinguish from the others standing or sitting at the end of the walkway. Only the keypoints of the legs were used, and the likelihood of leg keypoints had to be ≥0.6 to consider the frame for pose extraction. The location of the feet (on the ground or lifted to maximum) during the phases of walking was detected using the local maxima and minima of the keypoint coordinate time series data. Double support phases within bird and age were selected to minimize the vertical difference between the left and right feet. Maximum leg lifts during left and right steps were selected to maximize the vertical difference between the feet. For each bird-age-pose combination, maximum 3 frames per pose were considered for pose feature calculation, where the average of each feature over the 3 frames represented the feature of the bird at the given pose at the given age. The quality of automatically detected poses was checked visually by extracting the respective frames of 10 randomly selected birds from each age.

### Pose Features

**Figure 2:**
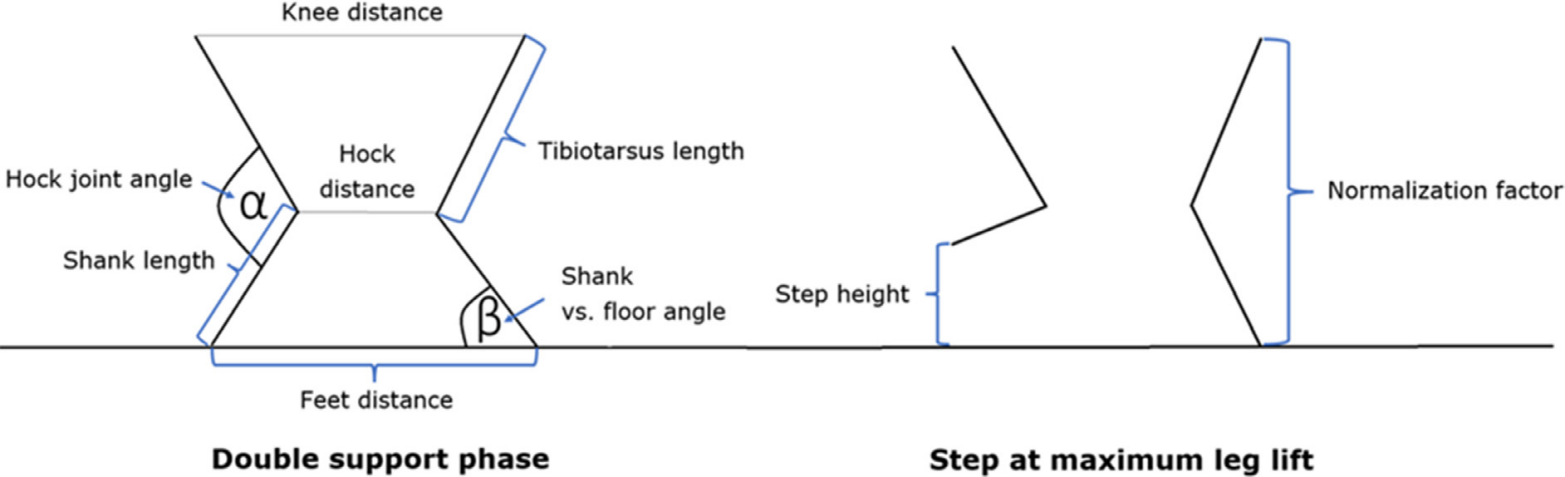
Schematic representation of the analyzed leg features at double support (left) and steps (right).

Altogether, we defined 7 features, 6 of which were quantified during the double support phase and 1 during the steps (Figure 2). Features during double support included hock joint lateral angles (degrees), medial angle of the shank and the horizontal line (degrees), normalized tibiotarsus length, normalized shank length, hock-knee distance ratio (the ratio between the horizontal distance of hocks and that of the knees), and hock-feet distance ratio (the ratio between the horizontal distance of hocks and that of the feet). The feature of interest during steps was the normalized step height (%, Figure 2). Tibiotarsus length, shank length, and step height were expressed in relation to a normalization factor, that is, the vertical difference between the highest knee and the lowest foot.

### Gait Scoring

The gait of the broilers at 33 d of age was assessed based on the recorded videos on a 0 to 5 scale, using the scoring system described in van der Sluis et al. (2021). Although this scoring system was not exactly the same as the one developed by Kestin et al. (1992), the overall idea is very similar, and the gait score categories from both scoring systems are assumed to represent similar gaits. Score 0 in the current scoring system represents birds walking very well, score 1 means good walking capabilities (controlled walk, capable of standing straight on legs), score 2 is a relatively good, oriented walk, score 3 is a mediocre walk where the bird is more out of balance, can translocate well but sits down quickly, score 4 is a poor walk (bent or spread legs, waddling, legs pointing outward, wings often hanging down), and broilers with score 5 can barely walk and also use their wings when walking. Gait scoring of all birds was performed by 4 experienced scorers (scorer A–D) independently. Each bird was assigned the mean of the independent gait scores. The same 15 birds were randomly selected for repeated gait assessment to test intraobserver reliability of each scorer. Due to the small sample size in some gait score categories, broilers were further classified into having either good (mean gait score ≤2) or suboptimal (mean gait score >2) gait. This cutoff value assumed that gait scores above 2 potentially lead to impaired welfare in broilers (Kestin et al., 1992; van der Sluis et al., 2021). To test the robustness of our conclusions, we re-ran the analyses with different mean gait score cutoff values (≤2.5 vs. >2.5 and ≤3 vs. >3), as well.

### Statistical Analyses

Statistical analysis was performed in R version 4.2.2 (R Core Team, 2022). We analyzed those birds that were present during the entire study (i.e., until d 33 of age). Therefore, our final dataset contained the pose feature data and body weight of 84 birds from the 3 ages and the corresponding gait scores at 33 d of age. For each combination of age and pose, pose features on either side lower than the lower quartile minus 2 times the interquartile range or higher than the upper quartile plus 2 times the interquartile range were excluded from the analyses to remove outliers. The correlations among the features of interest at 33 d of age were estimated using Pearson's correlation coefficient. Interobserver agreement among the gait scorers (on a scale 0–5) was assessed by Fleiss’ kappa, using the *irr* package (Gamer et al., 2019). Intraobserver reliability of the gait scores was estimated by Cohen's weighted kappa, using the *irr* package. The value of kappa (*κ*) can range between −1 and 1, and the strength of agreement can be interpreted as follows: *κ* < 0.00 = poor, *κ*: 0.00 to 0.20 = slight, *κ*: 0.21 to 0.40 = fair, *κ*: 0.41 to 0.60 = moderate, *κ*: 0.61 to 0.80 = good, *κ*: 0.81 to 1.00 = excellent agreement (Landis and Koch, 1977). The difference in body weight between broilers with good vs. suboptimal gait was analyzed using a generalized estimating equations model from the *geepack* package (Højsgaard et al., 2006), with age, binary gait class (good or suboptimal on d 33), and their interaction as fixed effects, and chicken ID as the grouping variable. Differences in pose features at 33 d of age between the 2 gait classes were analyzed using linear models with one of the pose features as the dependent variable, and gait class (good or suboptimal on d 33), body weight, side of the feature (left or right), and the interaction of gait class and side of the feature as explanatory variables. Body weight and side were included in the models to control for their (possible) effects on the values of the features. If the interaction between gait class and side of the feature was not significant, the interaction term was removed, and the model was refitted. None of the interactions between gait class and side were significant (all *P* > 0.05). Features without left and right counterparts (hock-knee distance ratio and hock-feet distance ratio) were analyzed using similar linear models, but with only gait class and body weight as explanatory variables. The level of significance was set to *P* ≤ 0.05, and 0.05 < *P* ≤ 0.10 was considered a tendency.

## RESULTS

### Pose Estimation

At 100,000 iterations, the train and test errors of the model were 2.12 px and 4.83 px, respectively. When only the filtered keypoints (i.e., keypoints with a likelihood ≥0.6) were considered in the error calculation, train and test errors were 2.11 px and 4.02 px, respectively. The percentage of frames exceeding the likelihood threshold decreased with age (Supplementary Figure 1), but overall, the confidence of the deep learning model in locating the body parts during the walking tests was high. Especially the leg keypoints, which were the main focus of our analyses, had high mean percentages of high-likelihood frames, with more than 70% of all recorded frames exceeding a likelihood threshold of 0.6 irrespective of age, on average.

### Pose Features

**Figure 3:**
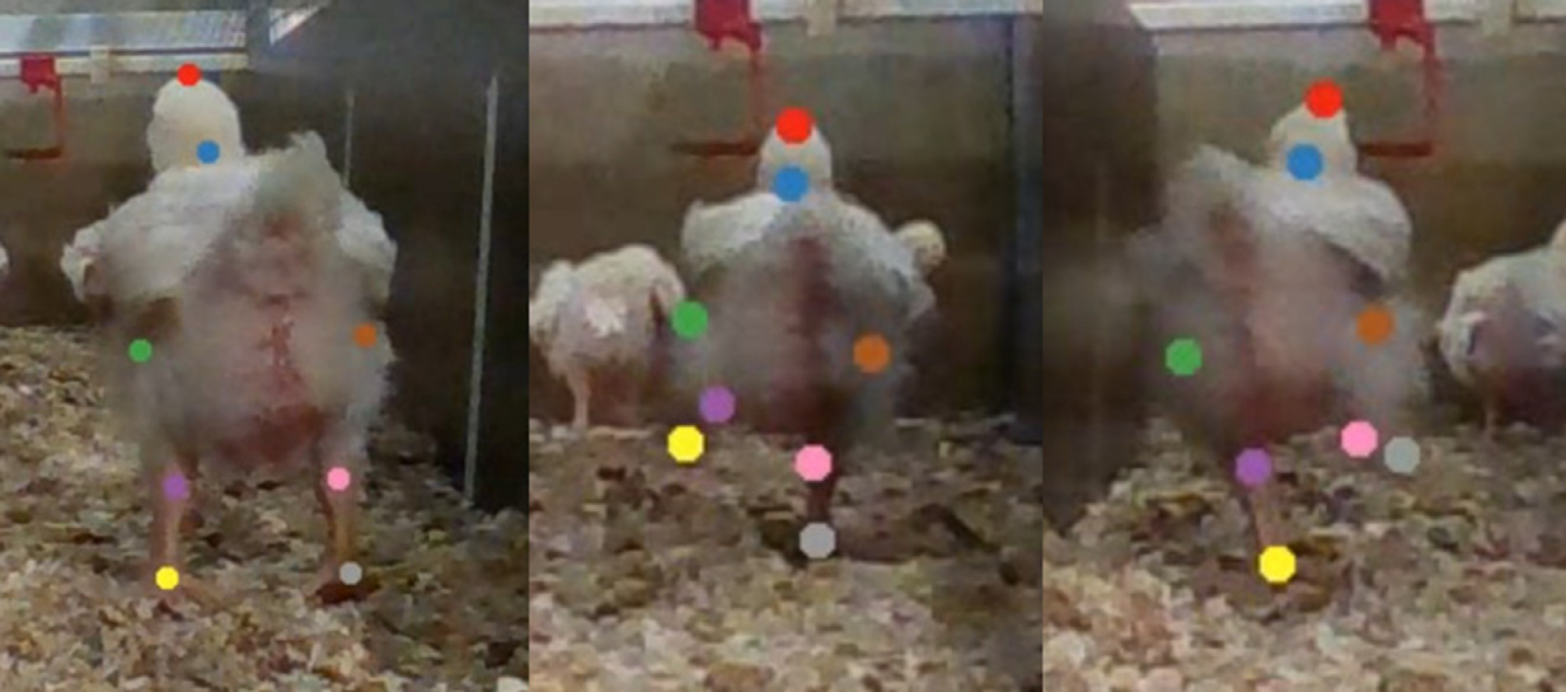
Automatically extracted double support stance, left and right steps on d 14 (images cropped and enlarged).

**Figure 4:**
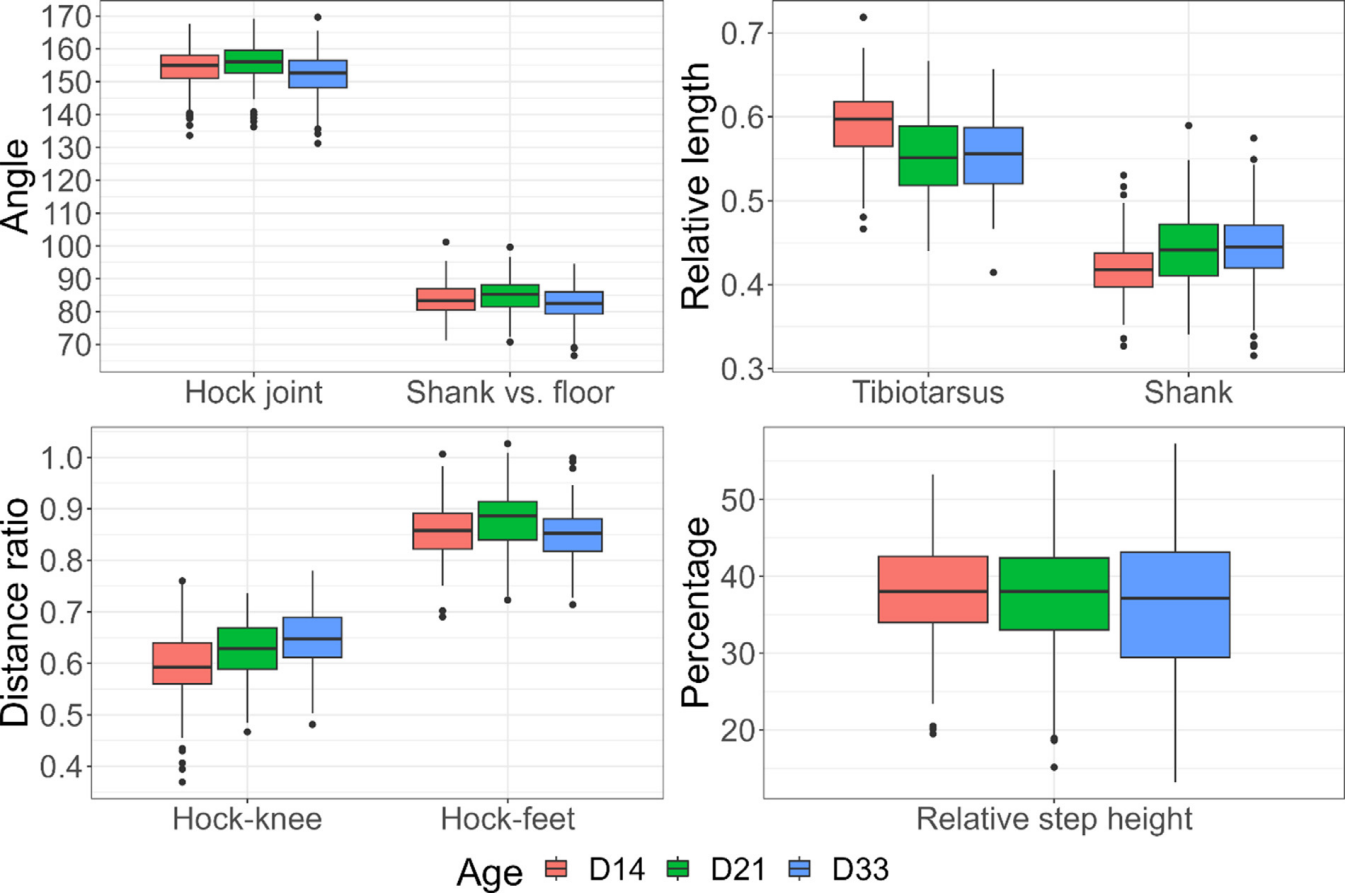
Boxplot of pose features by age.

Examples of extracted poses are shown in Figure 3. Summary statistics of pose features by age are shown in Table 1 and Figure 4. For most pose features, no clear trends were observed by age. However, hock-knee distance ratio increased slightly with age, meaning that the knees moved relatively further apart than the feet as the birds grew.

**Table 1:** Descriptive statistics of pose features by age.

Feature	D14			D21			D33		
	Mean	SD	*n*^1^	Mean	SD	*n*	Mean	SD	*n*
Hock joint angle (°)	154.2	6.2	164	155.6	6.0	164	151.9	6.9	164
Shank vs. floor angle (°)	83.9	5.0	166	84.9	5.0	162	82.4	5.8	163
Tibiotarsus relative length	0.59	0.04	158	0.55	0.05	165	0.55	0.05	168
Shank relative length	0.42	0.04	166	0.44	0.04	165	0.45	0.05	167
HKDR^2^	0.59	0.08	80	0.62	0.06	83	0.65	0.06	83
HFDR^3^	0.86	0.06	81	0.88	0.06	82	0.85	0.06	79
Relative step height (%)	37.9	6.8	167	37.5	7.2	166	35.9	9.6	161

1 Number of records.

2 Hock-knee distance ratio.

3 Hock-feet distance ratio. HKDR and HFDR do not have left and right counterparts, hence the number of records is lower for these features.

The correlations between the features of interest (step height during steps and the rest of the features during double support) were further analyzed at 33 d of age. Strong positive correlation was found between hock joint angle and shank vs. floor angle (0.75, *P* < 0.001). This implies that individual broilers tend to have either a more crouched or a straightened-up posture affecting both leg angles, rather than independently varying hock and shank angles. Strong negative correlation was detected between relative tibiotarsus length and relative shank length (−0.58, *P* < 0.001), which was expected, as both were expressed as relative values to the same normalization factor. Hock-feet distance ratio seems to capture angle information well, as it showed a strong positive correlation with leg angles (hock joint angle: 0.78, shank vs. floor angle: 0.90, both *P* < 0.001).

### Gait Scores

**Figure 5:**
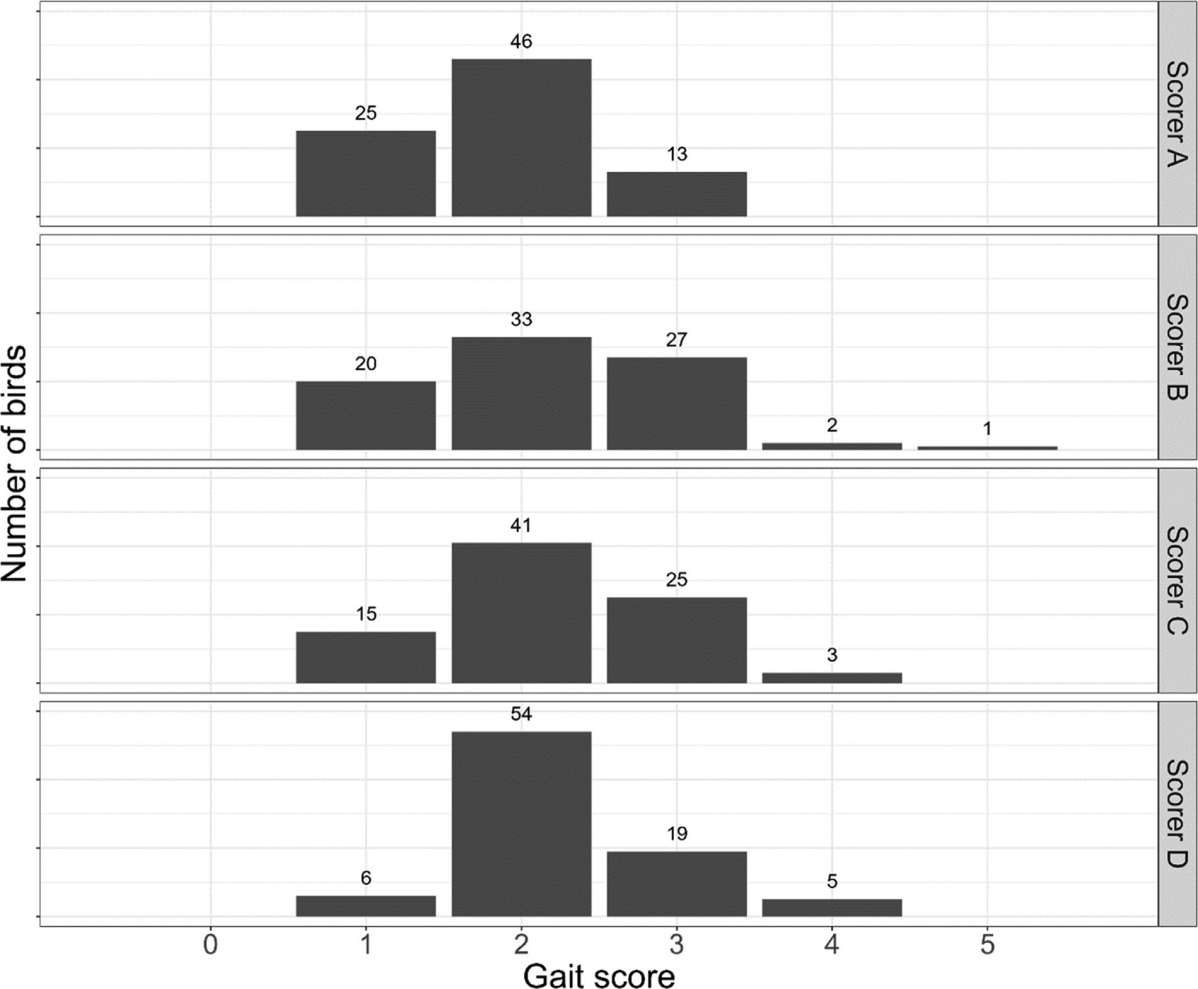
Distribution of the gait scores by observer.

The gait score results of the 84 birds included in our analyses are shown in Figure 5. On a scale from 0 to 5, none of the birds was assigned score 0, but also relatively few birds with very poor gait were found. The 3 birds excluded from the analyses because they did not walk during the walking test on d 33 had mean gait scores ≥4.25 (mean of scorers A–D), and these birds had the highest mean gait scores among all birds (data not shown). The highest mean gait score in the remaining sample of broilers was 3.75, which also implies that our findings do not represent birds with the poorest gait (as expected, because of the difficulties in obtaining proper poses). On the other hand, this also implies that our findings represent birds up to approximately gait score 4. Intraobserver agreement of the 4 scorers ranged between 0.67 and 1 (Cohen's weighted *κ*, good-excellent agreement). The value of Fleiss’ *κ* was 0.27, representing a fair interobserver agreement among the 4 scorers. After creating binary classes using mean gait score 2 as a threshold, 48 birds (57.1%) were classified as having good gait and 36 birds (42.9%) as having suboptimal gait.

### Relationship of Features With Body Weight and Walking Ability

Broilers with a suboptimal gait by 33 d of age were numerically heavier on each measurement day during their lifetime. The differences remained nonsignificant at 14 and 21 d of age, but a tendency for difference was found at 33 d of age. The body weight difference between birds with suboptimal vs. good gait was 9.7 g (95% CI: −12.3 to 31.7 g, *P* = 0.387) on d 14, 26.4 g (95% CI: −11.9 to 64.7 g, *P* = 0.177) on d 21, and 64.3 g (95% CI: −12.3 to 141.0 g, *P* = 0.100) on d 33. The body weight of the broilers by age and gait class is shown in Supplementary Figure 2.

The differences in the pose features on d 33 between broilers with good vs. suboptimal gait are shown in Table 2. Broilers with suboptimal gait had sharper hock joint angles during double support than broilers with good gait. The angle of the shank was also numerically sharper in broilers with suboptimal gait, but the difference was not statistically significant. Similarly, no statistically significant difference was found in relative tibiotarsus and shank lengths, and in hock-knee distance ratio. On the other hand, hock-feet distance ratio was lower in birds with suboptimal gait, that is, the feet of lame birds were relatively more spread out than the hocks compared to the broilers with good gait. Birds with suboptimal gait had lower relative step height, that is, they did not lift their feet as high during maximum leg lift as the good walkers. For each pose feature, the difference between gait classes was also expressed as a percentage, so the features can be compared to each other in terms of signaling gait problems (Table 2). Expressed in percentage difference, step height and hock-feet distance ratio showed the largest deviations between broilers with suboptimal vs. good gait. We also tested the robustness of our results against different gait score thresholds to distinguish broilers with good vs. suboptimal gait. The percentage difference between the resulting gait score classes for each feature is presented in Supplementary Figure 3. The main conclusions remained virtually unchanged despite the changes in gait score threshold.

**Table 2:** Difference in pose features on d 33 between broilers with good vs. suboptimal gait.

Feature	Value at good gait	Diff.^1^	95% CI	*P*	% diff.^1^	% diff.^1^ 95% CI
Hock joint angle (°)	152.9	−2.2	−4.3 to −0.1	0.042	−1.43	−2.82 to −0.05
Shank vs. floor angle (°)	82.9	−1.3	−3.1 to 0.6	0.174	−1.52	−3.72 to 0.68
Tibiotarsus relative length	0.55	0.01	−0.01 to 0.02	0.324	1.18	−1.18 to 3.55
Shank relative length	0.45	0.00	−0.01 to 0.01	0.945	0.11	−3.05 to 3.27
HKDR^2^	0.65	−0.01	−0.03 to 0.02	0.641	−1.02	−5.36 to 3.32
HFDR^3^	0.87	−0.03	−0.06 to −0.01	0.013	−3.62	−6.47 to −0.78
Relative step height (%)	37.7	−4.5	−7.2 to −1.7	0.002	−11.81	−19.16 to −4.46

1 Difference (suboptimal − good).

2 Hock-knee distance ratio.

3 Hock-feet distance ratio.

## DISCUSSION

In this study, it was investigated whether automatically detected poses could be used to characterize walking and lameness in broilers. In total, 7 pose features were successfully extracted from the videos and the relationships with manual gait scores were examined. Broilers that were manually classified as having suboptimal gait showed sharper hock joint angles, a lower hock-feet distance ratio and lower relative step heights. These differences between birds with good gait and birds with suboptimal gait have potential to be implemented in automation of gait scoring in broilers.

### Deep Learning Model Performance

Additional training of the existing model of Doornweerd et al. (2021) was needed to achieve a well-performing model for the current data collection environment. However, this required only a small additional dataset (181 frames), highlighting the added value of an existing model to build upon. Overall, the retrained pose detection model performed well, with train and test errors of 2.11 to 2.12 and 4.02 to 4.83, respectively, depending on whether all keypoints or only filtered keypoints were included. These values are in a similar range to those of Doornweerd et al. (2021), who reported values of 2.26 and 7.56, respectively, for keypoints that surpassed the filtering. However, the 8 keypoints showed differences in how well they could be located, with the neck and knees being harder to locate, and for the neck the detection certainty deteriorated over time. Likely, the neck and knees were harder to detect because of the thick layer of feathers covering these areas. Similar observations were made by Doornweerd et al. (2021), who reported lower percentages of keypoints remaining (i.e., keypoints with a likelihood ≥0.6 over the total number of keypoints with a Euclidean distance) for the knees. Furthermore, we observed that birds tended to walk with their head down as they grew, which could have led to the head and especially the neck being occluded from the camera's viewpoint by the body, explaining the deterioration in detection of the neck as the birds aged. However, as our study focused on leg keypoints, the deterioration in the detection of the neck had no impact on the results.

### Gait Scores and Body Weight

We used gait scores from multiple experienced observers to improve the robustness of our gold standard for walking ability, and reduce the effects of the subjectivity of gait scoring. The use of additional diagnostic methods would have led to stronger conclusions on leg health. For example, Fernandes et al. (2012) used autopsy to assess the relationship of several leg disorders with gait. As our study was focused on the relationship of pose features with gait score as a measure of walking ability, and not leg health per se, the interpretability of our findings in relation to leg disorders is limited.

The gait score distribution observed here, with 57% of the broilers showing a good gait and 43% showing a suboptimal gait, is in a similar range to other reports in literature. For example, van der Sluis et al. (2021) reported in their study that 58% of the broilers had a normal gait (GS ≤ 2) and 42% had an impaired gait (GS ≥ 3), and Knowles et al. (2008) reported in their UK survey that 27.6% of birds showed GS3 or higher. The final range of gait scores reported here was based on the scores assigned by 4 experienced observers, by taking the mean of the 4 scores. Although the intraobserver agreement was high (Cohen's weighted *κ* of 0.67–1), representing good to excellent agreement, the interobserver score was somewhat lower (Fleiss’ *κ* of 0.27, a fair agreement). This reflects the challenge of objective manual gait scoring, and highlights the value of an objective automated scoring system.

Twelve broilers were removed from the study due to routine activities for the breeding program. Removal was based on body weight only, so that the removed broilers represent the weight distribution within a pen. Choosing the individually tagged broilers based on a list (instead of the catching them as they come to hand in the pen), and adjusting all analyses for body weight, minimized the risk of introducing bias into our results.

In the current study, it was observed that broilers with an impaired gait at 33 d of age were, numerically, but not statistically significantly, heavier at 14, 21, and 33 d of age. This relationship between gait and body weight (also earlier in life) in broilers has been reported earlier. For example, van der Sluis et al. (2021) examined gait scores at 33 to 35 d of age, for broilers that had a relatively low or high body weight at approximately 2 wk old. They reported that within the lightweight broilers approximately 70% had GS ≤ 2, while within the heavyweight broilers approximately 46% had GS ≤ 2. This suggests that birds that are relatively heavy earlier in life might be more likely to develop gait problems later in life. Kestin et al. (2001) compared 13 broiler genotypes with 2 different feeding regimes and observed that the faster-growing genotypes had higher gait scores than slower-growing genotypes with the same diet. Moreover, when correcting for liveweight differences, the gait score of broilers was worse at 54 d than at 81 d. In other words, when birds gained body weight faster their gait score was worse (Kestin et al., 2001). The observations in the current study are in line with these earlier reports, and in the subsequent analyses body weight was therefore taken into account.

### Pose Estimation Features in Relation to Gait

Different broiler lines may differ in their posture while walking. For example, Paxton et al. (2013) aimed to quantify the general locomotor dynamics of modern broilers, with exclusion of visibly lame birds or birds that were incapable of sustained walking, using kinematic and force plate data. They observed that one of the pure lines included in their study showed a more crouched limb posture (based on a lower center-of-mass height for the same total leg length) than the other pure line and commercial line broilers. The current study indicates that there are also posture differences within a single broiler line, that have potential for automated scoring of gait, as our analysis revealed differences between birds with good gait and birds with suboptimal gait for several pose features.

Sex may also affect walking characteristics in broilers. Bokkers and Koene (2002) used fast-growing male and female broilers to study the ability to walk for a food reward. They found that males walked faster than females, despite their higher body weight, indicating differences in walking characteristics by sex in broilers. It is possible that after adjusting for body weight and gait, pose features would still differ between male and female broilers. As this study was a first attempt to link certain pose features to gait as a proof of concept, further work is necessary to optimize pose estimation for practical circumstances, and investigate the utility of pose features for gait assessment in different sexes, broiler lines, or even in other poultry species.

In terms of angle features, sharper hock joint angles were observed for broilers with suboptimal gait, indicating a more sagging posture. Moreover, a numerically sharper shank-vs.-floor angle was observed for broilers with suboptimal gait. These 2 features were observed to be strongly correlated, implying that individual broilers tend to have either a more crouched or a more straightened-up posture affecting both these leg angles simultaneously. Mendes et al. (2016) also studied leg angles in relation to gait scores in broilers, through the use of frontal and side-view photogrammetry. They examined the angle between the distal portion of the tibia and the center of the foot on the lateral side of the leg (which can be viewed as the supplementary angle of our shank-vs.-floor angle), and concluded that a larger lateral angle is correlated with worse gait in male broilers. In the current study, the medial shank-vs.-floor angle was sharper in broilers with an impaired gait, therefore, our results are in line with those of Mendes et al. (2016), although, the differences remained nonsignificant. Overall, it appears that hock joint angles, and perhaps shank-vs.-floor angles as well, have potential as indicators of gait impairment in broilers.

A difference in hock-feet distance ratio was also observed between birds with good gait and broilers with suboptimal gait, with broilers with suboptimal gait showing a smaller ratio. In other words, the feet of these birds were relatively more spread out than the hocks compared to the broilers with good gait. This might be linked to the sharper hock joint and shank-vs.-floor angles, as there were strong positive correlations between these angles and the hock-feet distance ratio. A smaller ratio could potentially indicate valgus deformations. It has been reported that gait problems and varus/valgus deformations are correlated (Sanotra et al., 2001). Fernandes et al. (2012) studied the relationship between different locomotion characteristics and observed that, at 35 d of age, there were positive correlations between valgus deformations in the left or right leg and gait scores. This appears to support the observation in the current study. However, it is important to note that the hock-feet distance ratio measured here may also simply be indicative of how birds are standing, for example, with X-shaped legs. Therefore, a smaller or larger hock-feet distance ratio does not automatically indicate valgus or varus deformations. However, if a small hock-feet distance ratio is detected by the pose estimation, this could contribute to a targeted approach where a human observer can double-check if valgus deformations are present.

In addition, it was observed that broilers with suboptimal gait had lower relative step heights, in line with earlier reports in literature. Caplen et al. (2012) studied the gait of lame (GS3) and nonlame (GS0) broilers using kinematic analysis. They observed, among other things, shorter stride lengths, higher lateral back displacement values (i.e., the maximum lateral back movement recorded during a stride), and lower vertical leg displacements (i.e., the maximum height that a leg is lifted during a stride) for the GS3 broilers. It appears that relative step height could be a useful indicator of lameness in broilers, especially given that, along with hock-feet distance ratio, step height showed the largest percentual difference between birds with good vs. suboptimal gait. Tracking these pose features over time along with the gait of broilers could enable timely detection of changes in individual walking characteristics, in order to predict the onset and progression of lameness.

### Toward the Future

The results from this study provide more insight into the differences in walking characteristics between broilers with good gait and broilers with suboptimal gait. Knowledge of these differences can be helpful in the design of automated gait score prediction models for broilers, and improve our understanding of some of the features that black box gait prediction models might pick up on. Currently, due to the limited data available, birds with different gait scores were grouped into good and suboptimal gait, using GS2 as the upper threshold for birds with good gait. This cutoff value was based on the general assumption that birds with GS higher than 2 have a potentially impaired welfare (Kestin et al., 1992), and was also used in other studies (e.g., van der Sluis et al., 2021). However, it has been reported that there is not a very clear distinction between birds with GS2 and birds with GS3 in terms of welfare (Skinner-Noble and Teeter, 2009), and there may be substantial differences between birds with GS0 and GS2 in locomotor ability (Tahamtani et al., 2021). The analyses in this study were also performed with GS 2.5 or 3 as cutoff values and did not result in substantially different conclusions. Nevertheless, additional research with larger numbers of broilers per gait score category could shed more light on the more subtle differences between different gait scores and may provide further input for future automation of gait scoring in broilers. A limitation of this study is that our automated pose estimation approach in its current form was not able to score walking ability of birds with very poor gait that do not walk in the walking test. However, future research could investigate the potential of implementing the automated pose estimation approach in environments where birds can roam freely, without using a walkway. In such an environment, it may be possible to capture on video even the lamest birds walking, for example, on their way toward drinkers or feeders, as vital resources. Whether it is then possible to reliably determine their walking ability using our pose estimation approach remains to be investigated.

## Appendix Supplementary materials

Image, application 1

## Footnotes

Supplementary material associated with this article can be found in the online version at doi:10.1016/j.psj.2023.102787.
