# Supplementary material for: Automated pose estimation reveals walking characteristics associated with lameness in broilers
Source: Poult Sci. 2023 May 19;102(8):102787. doi: 10.1016/j.psj.2023.102787 (PMC10404698; doi:10.1016/j.psj.2023.102787)
Supplement: Supplementary file 1 [file mmc1.docx]

Supplementary figure 1: Percentage of frames with keypoint likelihood exceeding 0.6, per body part and age (mean±SD, D: day, L: left, R: right)


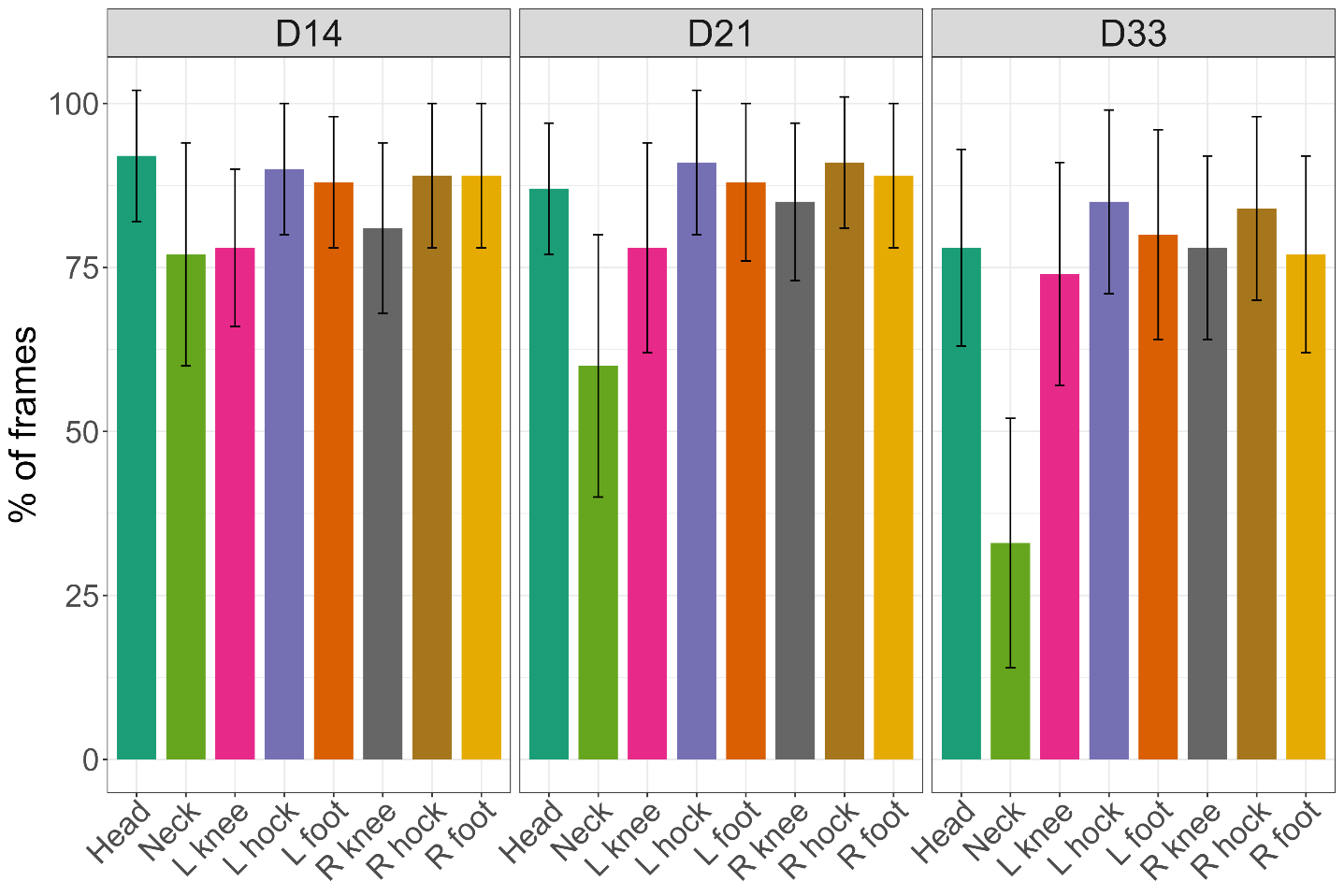


Supplementary figure 2: The body weight of broilers by age and gait class


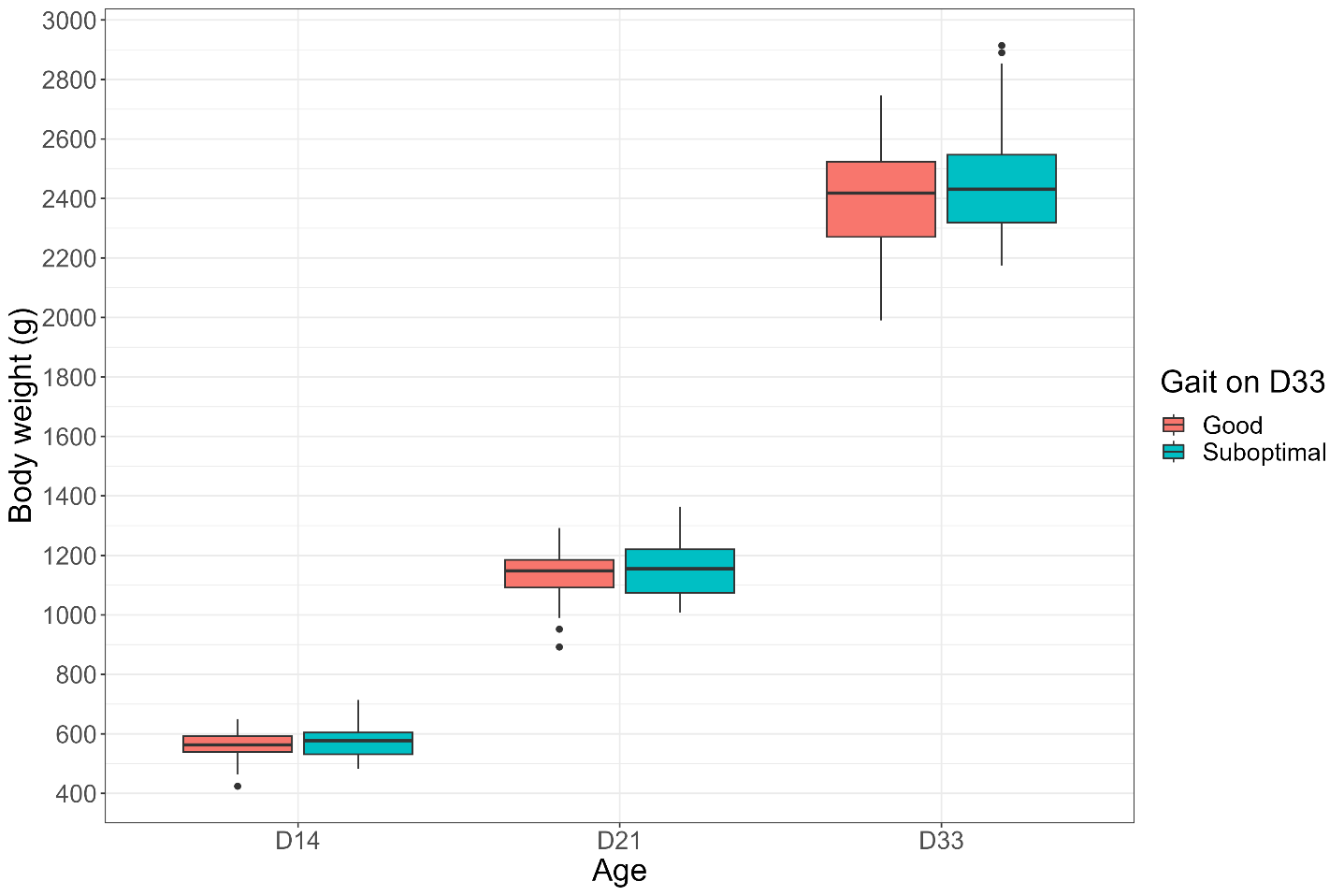


Supplementary figure 3: Percentage difference in pose features between broilers with good vs. suboptimal gait using different gait score thresholds (sensitivity analysis)


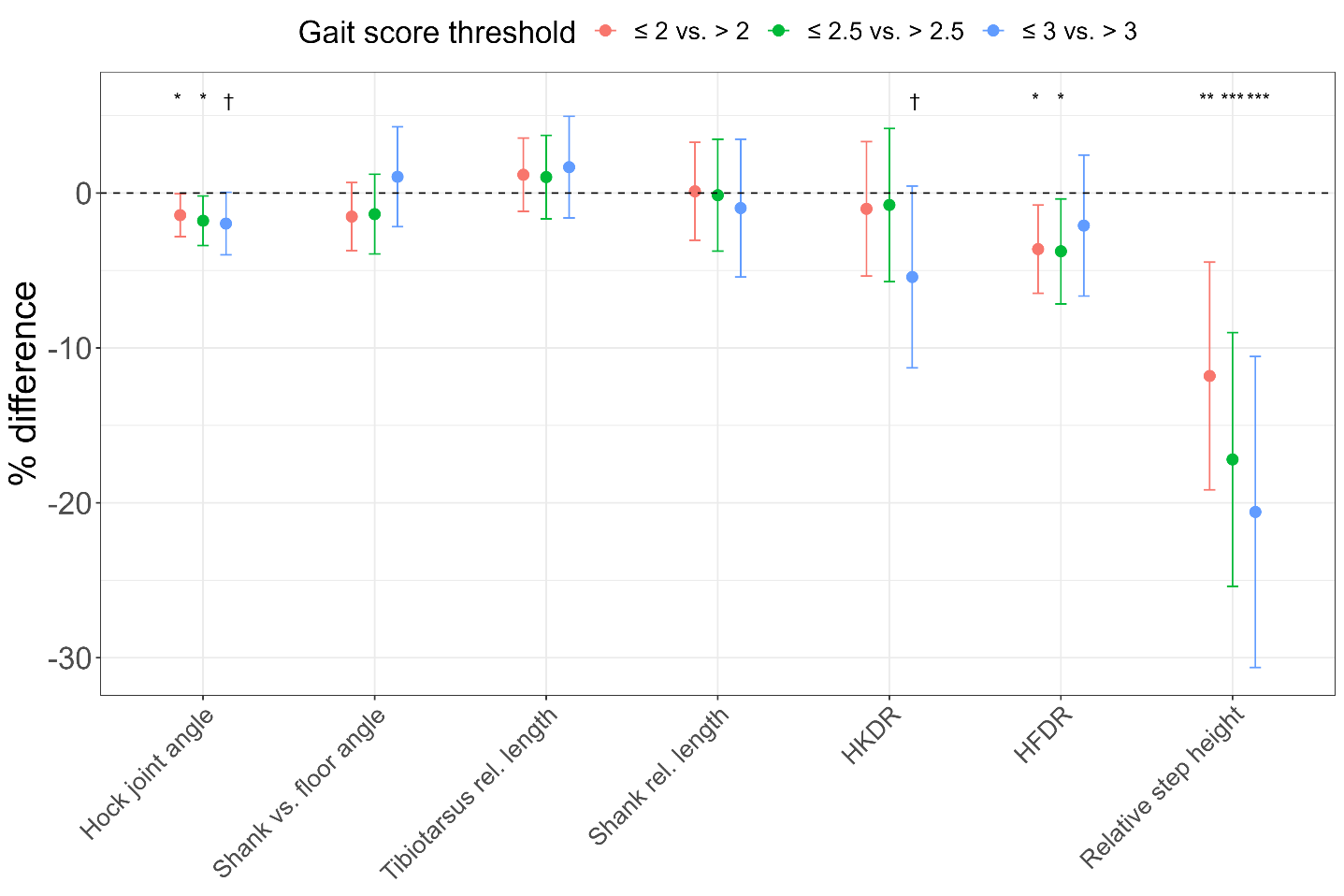


*P ≤ 0.05, **P ≤ 0.01, ***P ≤ 0.001, and †P ≤ 0.10
